# Borna Disease Virus 1 as Cause of Fatal Meningoencephalomyelitis in Wild Hedgehogs, Germany, 2022–2025

**DOI:** 10.3201/eid3205.250952

**Published:** 2026-05

**Authors:** Effrosyni Michelakaki, Benjamin Schade, Brigitte Boehm, Eva Kappe, Marcel Suchowski, Anne Kupca, Magdalena Schumacher, Anna Maria Gager, Friederike Liesche-Starnecker, Sonja Fiedler, Eva Schwarz, Zoltan Bago, Andreas Blutke, Martin Beer, Dennis Rubbenstroth, Kaspar Matiasek

**Affiliations:** Centre for Clinical Veterinary Medicine, Ludwig Maximilians-Universität München, Munich, Germany (E. Michelakaki, S. Fiedler, E. Schwarz, A. Blutke, K. Matiasek); Bavarian Animal Health Service, Poing, Germany (B. Schade, B. Boehm, E. Kappe); Bavarian Health and Food Safety Authority, Oberschleißheim, Germany (M. Suchowski, A. Kupca, M. Schumacher, A.M. Gager); University Medical Center Ulm, Ulm University, Ulm, Germany (F. Liesche-Starnecker); Institute for Veterinary Disease Control, Mödling, Austria (Z. Bago); Institute of Diagnostic Virology, Friedrich-Loeffler-Institut, Greifswald-Insel Riems, Germany (M. Beer, D. Rubbenstroth)

**Keywords:** Borna Disease Virus 1, BoDV-1, viruses, meningitis/encephalitis, emerging, *Erinaceus europaeus*, hedgehog, zoonoses, insectivore, Eulipotyphla, host, lethal, endemic, Germany

## Abstract

Borna disease virus 1 (BoDV-1) causes encephalitis with a fatality rate of >90% in domestic mammals and humans. Currently, the bicolored white-toothed shrew is the only known reservoir host. We report BoDV-1 infections in 15 wild European hedgehogs from an endemic area in Germany. Because hedgehogs are distant relatives of shrews and often cared for by humans, the cases raise concern regarding a potential zoonotic risk. All the hedgehogs that tested positive for BoDV-1 died of neurological disease and exhibited severe polio-predominant lymphoplasmohistiocytic meningoencephalitis. However, because of the detection of viral antigens in nonneural cells in 1 animal, we cannot completely exclude that some infected hedgehogs shed the virus. Although direct BoDV-1 transmission is known to be inefficient, our results emphasize the necessity of hygiene measures when handling hedgehogs, especially those with neurological signs who are from BoDV-1–endemic regions.

Borna disease virus 1 (BoDV-1; species *Orthobornavirus bornaense*, family Bornaviridae) is known as the causative agent of Borna disease, a usually fatal, immune-mediated meningoencephalitis identified throughout endemic areas of Germany, Austria, Liechtenstein, and Switzerland (*1*). Borna disease can affect a broad range of domestic mammals, particularly horses, alpacas, and sheep (*1*–*4*). Since 2018, BoDV-1 has also been shown to cause encephalitis in humans. Up to 6 cases are reported each year, and the case-mortality rate is >90% (*1*,*5*–*7*). Domestic mammals and humans are known to serve as dead-end hosts, in which the virus possesses an almost exclusively neurotropic tissue distribution without detectable viral shedding (*3*,*8*). Those infections resulted from spillover transmission from a natural reservoir (*1*,*9*,*10*). Currently, the insectivorous bicolored white-toothed shrew (*Crocidura leucodon*) is the only known reservoir host species (*11*–*13*). Infected shrews develop lifelong viral persistence with a broad tissue distribution and continuous viral shedding but no apparent tissue lesions or clinical disease (*11*).

The European hedgehog *(Erinaceus europaeus*) is another insectivorous species indigenous to Europe that often comes into close contact with humans, particularly when being cared for during hibernation in private households and rescue centers. Although hedgehogs have been associated with various zoonotic diseases (*14*,*15*), we have been unable to find reports of hedgehog BoDV-1 infection.

We present a study of BoDV-1 infection and fatal encephalitis in 15 wild European hedgehogs from an endemic area in Germany. The first case was detected in 2022. BoDV-1 was identified by quantitative reverse transcription PCR (qRT-PCR) of brain tissue from infected hedgehogs. Previous testing for various encephalitic pathogens reported in this or other species, such as tickborne encephalitis virus (TBEV) (*16*), rabies virus (*17*), canine distemper virus (CDV) (*18*) and rustrela virus (RusV) (*19*), yielded negative results. After the diagnosis of a second case in May 2024, the awareness of BoDV-1 among hedgehog rescue centers and diagnostic institutions in the region increased considerably, which led to the identification of a series of additional cases.

The taxonomic proximity of hedgehogs with shrews (*20*), the known BoDV-1 reservoir hosts, raised concerns about hedgehogs being able to shed the virus, leading to potential human exposure. Therefore, we performed a comprehensive analysis of the confirmed cases, including detailed histopathologic study and extensive characterization of the viral tissue distribution and cell tropism by using immunohistochemistry (IHC), RNAscope (Advanced Cell Diagnostics, Inc., https://acdbio.com) in situ hybridization (ISH), and phylogeographic analysis of hedgehog-derived BoDV-1 sequences. In addition, we initiated a screening of nonencephalitic hedgehogs from endemic areas that is ongoing.

## Methods

### Case Selection, Sample Collection, and Diagnostic Investigations

Our investigation focused on 16 wild hedgehogs that died or were euthanized because of nonsuppurative encephalitis with a histopathologic diagnosis at postmortem examination. In addition, we included 33 deceased nonencephalitic hedgehogs from endemic regions in Bavaria as controls (Appendix Table 1, https://wwwnc.cdc.gov/EID/article/32/5/25-0952-App1.pdf).

All animals underwent a complete postmortem examination, and we fixed a broad set of organs and tissues in 10% neutral buffered formalin for >24 hours for histopathologic analysis. From most cases, we snap-froze brain tissue and additional samples for further investigation. In addition, we collected fecal samples, urine samples, and oral swab samples beginning with case 7.

We tested fresh-frozen or formalin-fixed paraffin-embedded (FFPE) brain tissue of all animals by using a BoDV-1–specific qRT-PCR for BoDV-1 RNA (*1*,*3*). We also tested all available fresh-frozen samples and swab samples of BoDV-1–positive animals.

For all BoDV-1–positive animals, we performed staining for BoDV-1 antigen and RNA by IHC and RNAscope ISH. In addition, we tested brain samples from encephalitic animals for other known encephalitic viruses, including TBEV and RusV by qRT-PCR (Appendix Table 2) (*21*,*22*), rabies virus (*23*), and CDV (*24*) by IHC (Appendix section B, Table 3).

### Screening for BoDV-1 Antigen Distribution by IHC

We conducted IHC for BoDV-1 nucleoprotein (N) by using mouse monoclonal antibody Bo18 (*25*) on all sections of all available tissues. We also used rabbit anti–BoDV-1 nucleoprotein polyclonal hyperimmune serum #201 (*3*) on select central nervous system (CNS) and peripheral nonneural tissue sections of case 5. All procedures are described in detail (Appendix section B, Table 3).

### Screening for BoDV-1 RNA Tissue Distribution via RNAscope ISH

We conducted RNAscope ISH on tissue sections of the CNS of all BoDV-1–positive animals and a selection of peripheral organs for BoDV-1 RNA (probe V-BoDV1-G targeting viral RNA encoding for the matrix protein and glycoprotein genes; genome positions 2,236–3,747 of BoDV-1) (GenBank accession no. NC_001607.1). We conducted ISH as described previously (*26*).

### Lesion Characterization

We macroscopically evaluated formalin-fixed tissues before and after fixation and trimming. We trimmed the brain at multiple planes and processed representative areas of telencephalon, diencephalon, brain stem, and cerebellum for microscopic examination. Spinal cord sections comprised transverse and longitudinal sections upon decalcification of the vertebral column by a 20% EDTA solution. We took representative sections from all available adequately preserved peripheral organs and tissues. All samples underwent an ascending alcohol series up to xylene by using an automatic histoprocessor. Thereafter, we embedded the samples in paraffin, cut into 2–4 µm–thick sections, and then stained all sections with hematoxylin and eosin stain for routine microscopic examination (*27*).

To phenotype the inflammatory cell infiltrations, we performed IHC by staining for T lymphocyte marker CD3, B lymphocyte marker Pax 5, and macrophage and microglial marker Iba1. We highlighted the degree and distribution of gliosis by using the astrocyte marker glial fibrillary acidic protein (GFAP). All procedures including detailed information on used antibodies are described (Appendix).

### RNA Extraction and qRT-PCR Testing for BoDV-1

We extracted RNA from fresh-frozen tissue samples and swabs by using the NucleoMag VET kit (Macherey-Nagel, https://www.mn-net.com) with a KingFisher Flex Purification System (Thermo Fisher Scientific, https://www.thermofisher.com), whereas we used the RNeasy FFPE kit (QIAGEN, https://www.qiagen.com) for FFPE tissue, as described previously (*1*). We conducted semiquantitative detection of BoDV-1 RNA by qRT-PCR BoDV-1 Mix-1 and Mix-6 (Appendix Table 2), as described previously (*1*,*7*). We compiled qRT-PCR results as cycle quantification (Cq) values. We used an RNA preparation of BoDV-2 isolate number 98 (GenBank accession no. AJ311524.1) as a positive control and for calibration of the Cq values.

For all BoDV-1-positive animals, we determined partial BoDV-1 genome sequences covering at least the N, accessory protein, and phosphoprotein genes (1,824 bases, positions 54 to 1,877) (GenBank accession no. U04608.1) by Sanger sequencing of overlapping conventional reverse transcription PCR products, as described previously (*1*,*3*). BoDV-1 sequences generated in this study were deposited in GenBank (accession nos. PV357162.1–8.1 and PZ000771.1–8.1). We performed phylogenetic analysis by using Geneious Prime version 2021.0.1 (Geneious, https://www.geneious.com). We calculated a neighbor-joining tree by using the Jukes-Cantor model of all 15 hedgehog-derived BoDV-1 sequences together with 258 N-X/P sequences from naturally infected animals and humans available from public databases (*1*,*9*). We used the sequence of isolate BoDV-2 No/98 (GenBank accession no. AJ311524.1) to root the tree.

## Results

### Diagnostic Testing, Time of Appearance, Geographic Origin, and Clinical Manifestation

Sixteen of the 49 evaluated hedgehogs from Bavaria demonstrated lymphoplasmohistiocytic meningoencephalitis. BoDV-1 RNA was detected in the brains of 15 of those animals (Table) but not in any of the 33 nonencephalitic hedgehogs (Appendix Table 1). Differential diagnostic testing by IHC and qRT-PCR did not detect TBEV, RusV, CDV, or rabies virus in any of the encephalitis cases (data not shown).

**Table:** Overview of BoDV-1–infected European hedgehogs included in study of Borna disease virus 1 causing fatal meningoencephalomyelitis in wild hedgehogs, Germany, 2022–2025

Case no.	District	Rescue center	Date found	Date died	Survival time after admission or disease onset*
1	Rottal-Inn	A	2022 Jun 22	2022 Jul 16	24
2	Ebersberg	Private	2024 Apr 30	2024 May 5	5
3	Rottal-Inn	A	2024 Jul 1	2024 Jul 10	9
4	Ebersberg	B	2024 Jun 24	2024 Jul 12	20
5	Rottal-Inn	Private	2024 Jun 23	2024 Jul 24	24
6	Ebersberg	B	2024 Jun 18	2024 Aug 15	58
7	Rottal-Inn	A	2024 Sep 11	2024 Sep 12	1
8	Landsberg am Lech	C	2025 Apr 9	2025 Apr 29	5*
9	Eichstätt	D	2025 May 14	2025 May 23	9
10	Eichstätt	D	2025 May 17	2025 May 23	6
11	Ebersberg	E	2025 May 16	2025 Jun 3	18
12	Rosenheim	F	End of 2024	2025 Jun 7	4*
13	Traunstein	F	2025 May 28	2025 Jun 10	13
14	Roth	G	2025 Jul 17	2025 Jul 19	2
15	Landsberg am Lech	Private	2025 Jul 26	2025 Jul 31	5

*Case 8 was reported to demonstrate first clinical signs on 2025 Apr 24. Case 12 had been submitted to the rehabilitation center already several months earlier and had hibernated there. Case 12 began demonstrating neurologic signs on 2025 Jun 3.

The first BoDV-1–infected animal died in July 2022, followed by 6 BoDV-1–positive cases during May–September 2024 and 8 cases during April–July 2025 (Table). All BoDV-1–positive hedgehogs originated from the known BoDV-1–endemic area in Bavaria; they were submitted for necropsy by different hedgehog rescue centers or by private persons (Table). At necropsy, the hedgehogs weighed 400–960 g. Eight were female and 7 were male. All cases, except case 8 and case 12, had already exhibited neurologic signs by the time they were found. Clinical manifestations developed in case 8 after 15 days of care, whereas case 12 had already been in the rehabilitation center for >6 months and hibernated there before neurologic signs developing in June 2025 (Table). According to the information provided by the submitters, clinical manifestations included incoordination, gait abnormalities, seizures, apathy, spontaneous muscle twitching, impaired thermoregulation, and vestibular signs with unilateral head tilt (Appendix Table 4). As the clinical signs progressed and the animals were not responsive to the administered treatments, all hedgehogs were euthanized because of poor prognosis or died during days 1–58 of care (Table).

### BoDV-1–Associated Lesion Patterns

**Figure 1:**
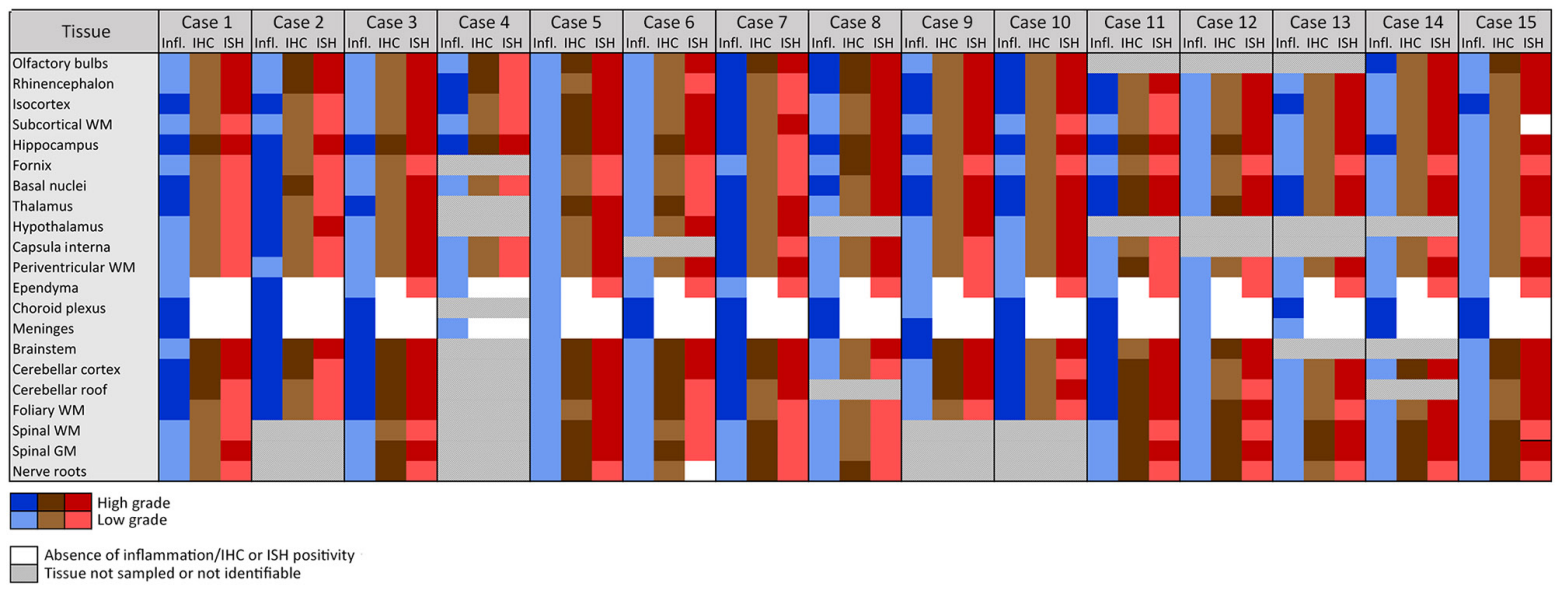
Inflammatory lesions and Borna disease virus 1 antigen and RNA detection in the central nervous system of infected European hedgehogs in study of the virus as cause of fatal meningoencephalomyelitis in wild hedgehogs, Germany, 2022–2025. GM, gray matter; IHC, immunohistochemistry; infl., inflammation; ISH, RNAscope in situ hybridization (Advanced Cell Diagnostics, Inc., https://acdbio.com); WM, white matter.

**Figure 2:**
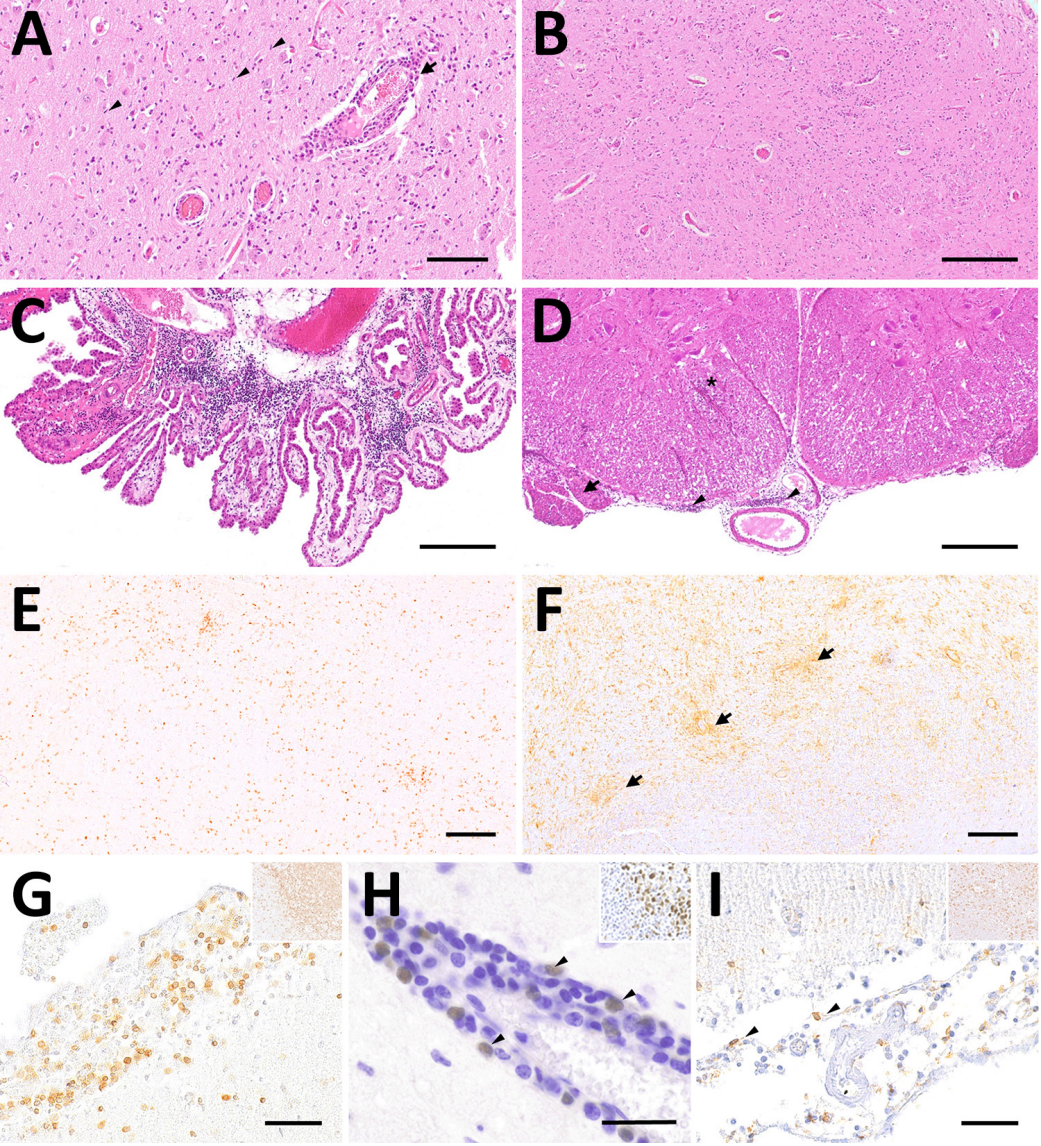
Representative changes and composition of immune cells in the central nervous system of Borna disease virus 1–infected hedgehogs in study of the virus as cause of fatal meningoencephalomyelitis in wild hedgehogs, Germany, 2022–2025. A) Multifocal lymphohistiocytic infiltrates with perivascular cuff formation (arrow) and microgliosis (arrowheads) from case 7. Scale bar represents 100 μm. B) Low-magnification section of brainstem from case 7 showing marked hypercellularity, with scattered lymphocytic infiltrates intermingled with astro- and microgliosis. Scale bar represents 250 μm. C, D) Infiltrates also extend into the choroid plexus in case 6 (C) and the subarachnoid space (arrowheads), nerve roots (arrow) and spinal white matter (asterisk) from case 5 (D). Scale bars represent 250 μm. E) CD3 immunohistochemistry of the corresponding brainstem region shown in Figure 1, panel B, confirms the widespread T lymphocyte infiltration, comprising the most invading immune cells. Scale bar represents 250 μm. F) The second most prominent cells involved in antiviral responses are Iba1-positive microglial cells and macrophages (arrows), as seen here in case 7. Scale bar represents 200 μm. G) Subarachnoid spaces contain numerous CD3-positive T lymphocytes in case 7. Scale bar represents 50 μm. H) Pax-5 positive B lymphocytes (arrowheads) resemble a minority of immune cells that are here seen concentrated around a blood vessel (arrowheads) from case 1. Scale bar represents 25 μm. I) Scattered Iba1-positive macrophages within subarachnoid spaces from case 7. Scale bar represents 50 μm. Insets in figures G, H, and I show the positive controls for each respective immunohistochemical staining (original magnification ×20). Stains: panels A–D, hematoxylin and eosin; E–I, 3,3′-diaminobenzidine, hematoxylin counterstain, immunohistochemistry using markers; E, CD3; F, Iba1; G, CD3; H, Pax5; I, Iba1.

No macroscopical alterations showed in the central or peripheral nervous system (PNS) of BoDV-1–infected hedgehogs. According to the histopathologic testing, the neurologic manifestations observed in all BoDV-1–positive animals included generalized angiocentric lymphoplasmohistiocytic meningoencephalitis (n = 4) or meningoencephalomyelitis (n = 11). The inflammatory infiltrates were widespread throughout all CNS regions of all 15 animals (Figure 1) and multifocally invaded the subarachnoid space, choroid plexus stromata, and neuroparenchyma (Figure 2, panels A–D). Neuronophagia and neuronal necrosis were observed in rare, scattered neurons. Moderate to marked, multifocal microglial activation and astrogliosis (highlighted by Iba-1 or GFAP staining) were most extensive in the gray matter of cerebral cortices, diencephalon, and brainstem, occasionally forming glial nodules (Figure 2, panels B, F). All cases featured mild to moderate intralesional edema.

IHC-based phenotyping of inflammatory cells revealed the affected hedgehog brains to be extensively infiltrated by CD3+ T lymphocytes, occasionally forming perivascular cuffs of up to 3–4-layer thickness (Figure 2, panels E, G), similar to other dead-end hosts, although more widespread (*28*–*30*). Inflamed zones also showed moderate numbers of Iba1-positive macrophages, activated Iba1-positive microglial cells (Figure 2, panels F, I) and GFAP-positive astrocytes, but only a few scattered and mostly perivascular Pax5-positive B cells (Figure 2, panel H). In addition, mild intraaxial vasculitic features were observed in cases 5 and 7.

The spinal cord was overall less severely affected, featuring multifocal lymphoplasmohistiocytic infiltration mostly within subarachnoid spaces (Figure 2, panel D). Of note, mild to moderate, multifocal infiltration and spongiosis of spinal white matter occurred in all animals, even in areas with spared gray matter (Figure 2, panel D). The inflammatory infiltrates also extended into adjacent nerve roots and dorsal root ganglia (Figure 1; Figure 2, panel D). Large fascicular nerves, distal, intramural ganglia and nerve branches showed minimal to no inflammation, except for mild to moderate, focally extensive, lymphocytic infiltration of the cranial mesenteric ganglia and nerves in case 3.

We did not observe intranuclear Joest-Degen inclusion bodies. We compiled information on concurrent pathologies in BoDV-1–positive hedgehogs (Appendix).

### Cell Tropism and Tissue Distribution of BoDV-1 RNA and Antigen

**Figure 3:**
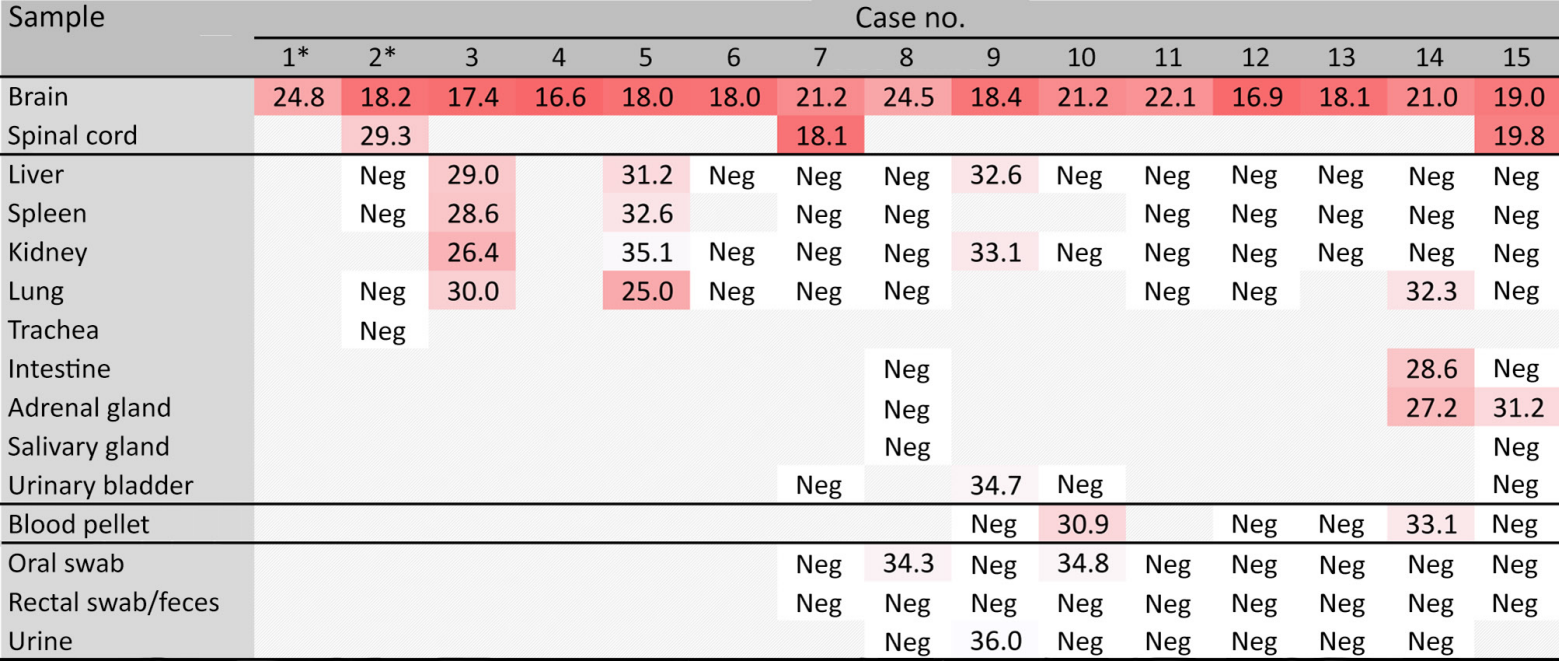
Detection of Borna disease virus 1 RNA by quantitative reverse transcription PCR in hedgehogs in study of the virus as cause of fatal meningoencephalomyelitis in wild hedgehogs, Germany, 2022–2025. Samples tested were available fresh-frozen neural and extra-neural tissues and additional samples collected postmortem. Results are presented as cycle quantification values. *Only formalin-fixed paraffin-embedded brain tissue was available for cases 1 and 2. Neg, negative.

We detected moderate to high levels of BoDV-1 RNA (Cq 16.6–24.8) in the brains of all BoDV-1–positive animals and in the spinal cord when available (Figure 3). Variable sets of fresh-frozen peripheral organs were available from 13 animals. Five animals had low to moderate BoDV-1 RNA levels (Cq 25.0–34.7) detectable in up to 4 peripheral tissue samples. Low levels of viral RNA (Cq 30.9–33.1) were also detectable in 2 of 6 blood samples collected (Figure 3). Postmortem oral swab, fecal, and urine samples were collected from 9 animals. Very low viral RNA loads (Cq 34.3–36.0) were detectable in 2 oral swab samples and 1 urine sample (Figure 3).

**Figure 4:**
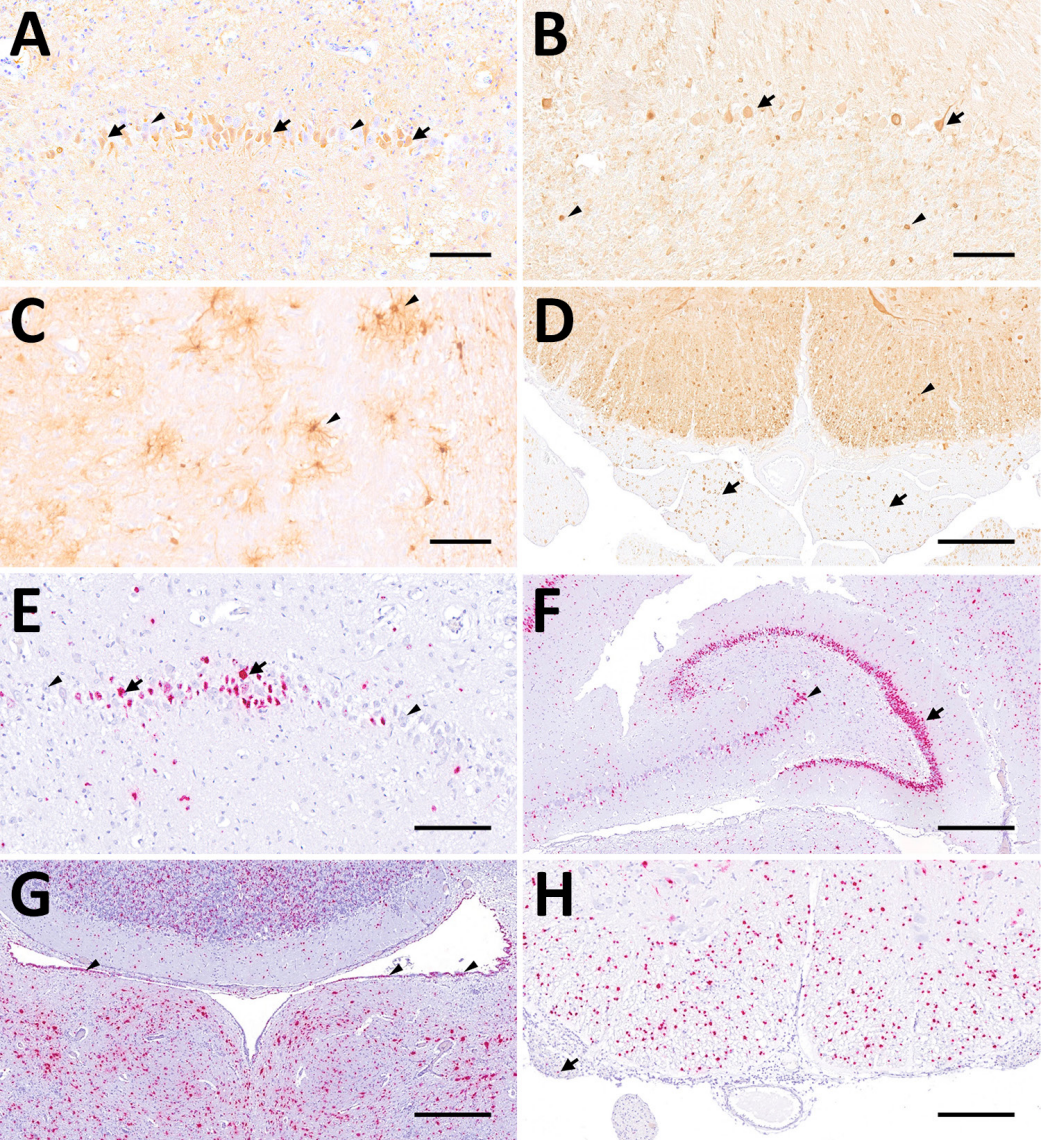
Distribution of Borna disease virus 1 detected within the CNS of infected hedgehogs in study of the virus as cause of fatal meningoencephalomyelitis in wild hedgehogs, Germany, 2022–2025. A–D) Antigen detected by immunohistochemistry by using Bo18 antibody. A) Multiple immunopositive neurons within the pyramidal layer of the hippocampus (arrows) mingling with negative neurons (arrowheads) from case 1. Scale bar represents 100 μm. B) Multiple immunopositive Purkinje (arrows) and few granule cells (arrowheads) within the cerebellar cortex from case 3. Scale bar represents 100 μm. C) Multiple positive astroglial cells within the spinal white matter (arrowheads) from case 6. Scale bar represents 100 μm. D) Multiple positive oligondendroglial cells within the spinal white matter (arrowhead), as well as multiple positive nerve fibers within the adjacent nerve roots (arrows) from case 5. Scale bar represents 250 μm. E–H) Virus RNA detected by RNAscope (Advanced Cell Diagnostics, Inc., https://acdbio.com) in situ hybridization (ISH). E) Numerous ISH-positive neurons (arrows) mingling with negative ones (arrowheads) within the corresponding area of the hippocampus shown in (A) from case 1. Scale bar represents 100 μm. F) Granule cells of dentate gyrus (arrow) are almost entirely ISH-positive as do multiple neurons of cornu ammonis (arrowhead) from case 5. Scale bar represents 500 μm. G) Numerous ISH-positive neurons, glial as well as ependymal cells (arrowheads) surrounding the fourth ventricle from case 7. Scale bar represents 500 μm. H) Numerous ISH- positive oligodendroglial cells within the spinal white matter and rare signals within nerve roots (arrow) from case 5. Scale bar represents 250 μm. Stains: A–I, 3,3′-diaminobenzidine with hematoxylin counterstain; E–H, 2.5 HD assay–RED with hematoxylin counterstain.

IHC using the Bo18 antibody and RNAscope ISH revealed widespread cytoplasmic and nuclear positivity for the BoDV-1 N protein and genomic RNA across neurons and glial cells without consistent hot spots in the brains and spinal cords of all BoDV-1–infected animals (Figures 1, 4). We observed almost diffuse reactivity throughout the neuroparenchyma, including white matter areas. IHC and ISH results agreed with each other, except for the ependymal layer, in which only RNA was detected in 4 animals (Figure 4, panel G). Neither antigen nor RNA were detected in choroid plexus and meninges (Figure 1).

**Figure 5:**
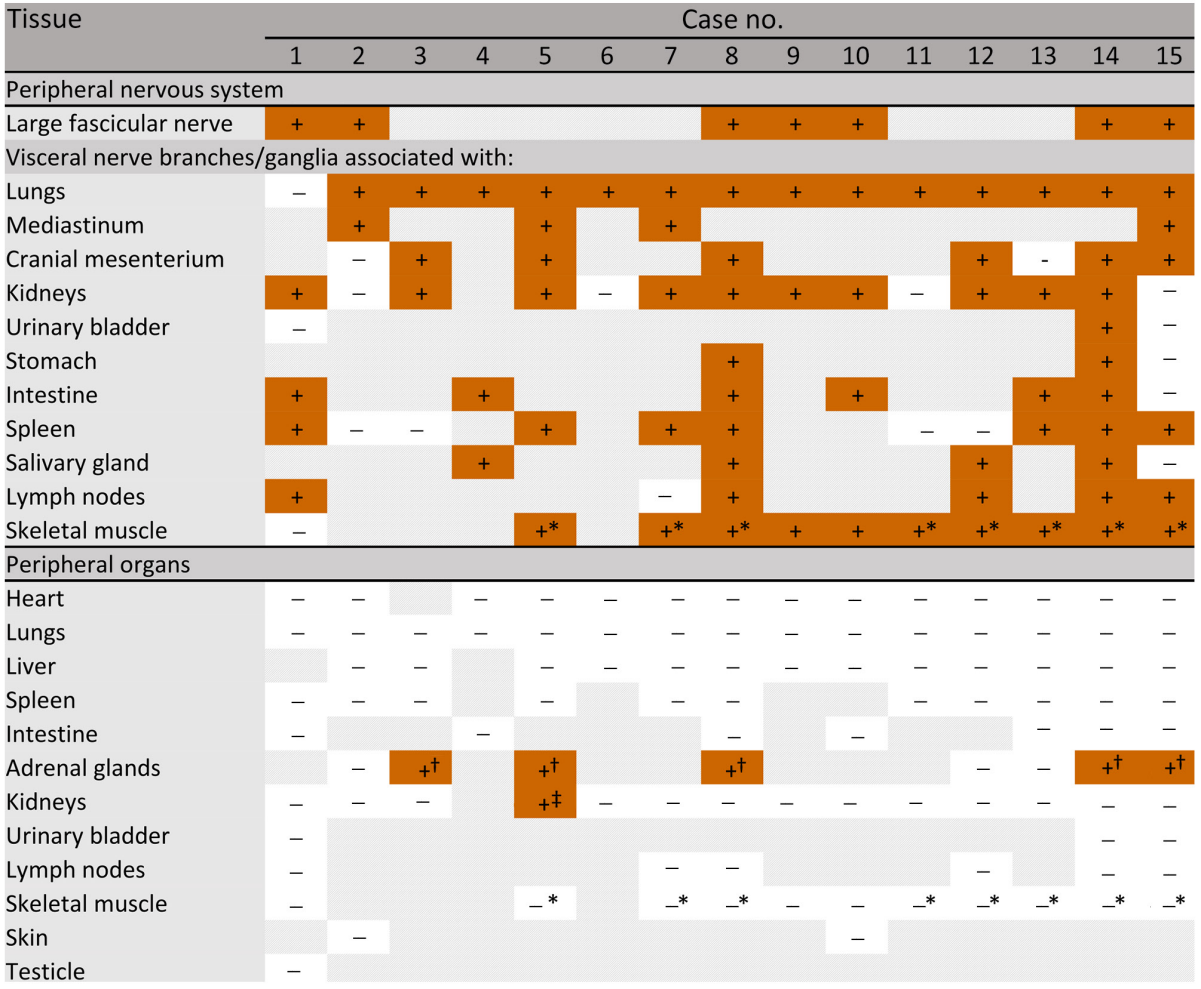
Borna disease virus 1 antigen detection by immunohistochemistry by using Bo18 antibody in peripheral nerves and peripheral organs of European hedgehogs in study of the virus as cause of fatal meningoencephalomyelitis in wild hedgehogs, Germany, 2022–2025. *Paravertebral musculature; †chromaffin cells; ‡1 focus of renal tubular epithelial cells. Nerve branches of organs that were not positive in any animal are not listed.

**Figure 6:**
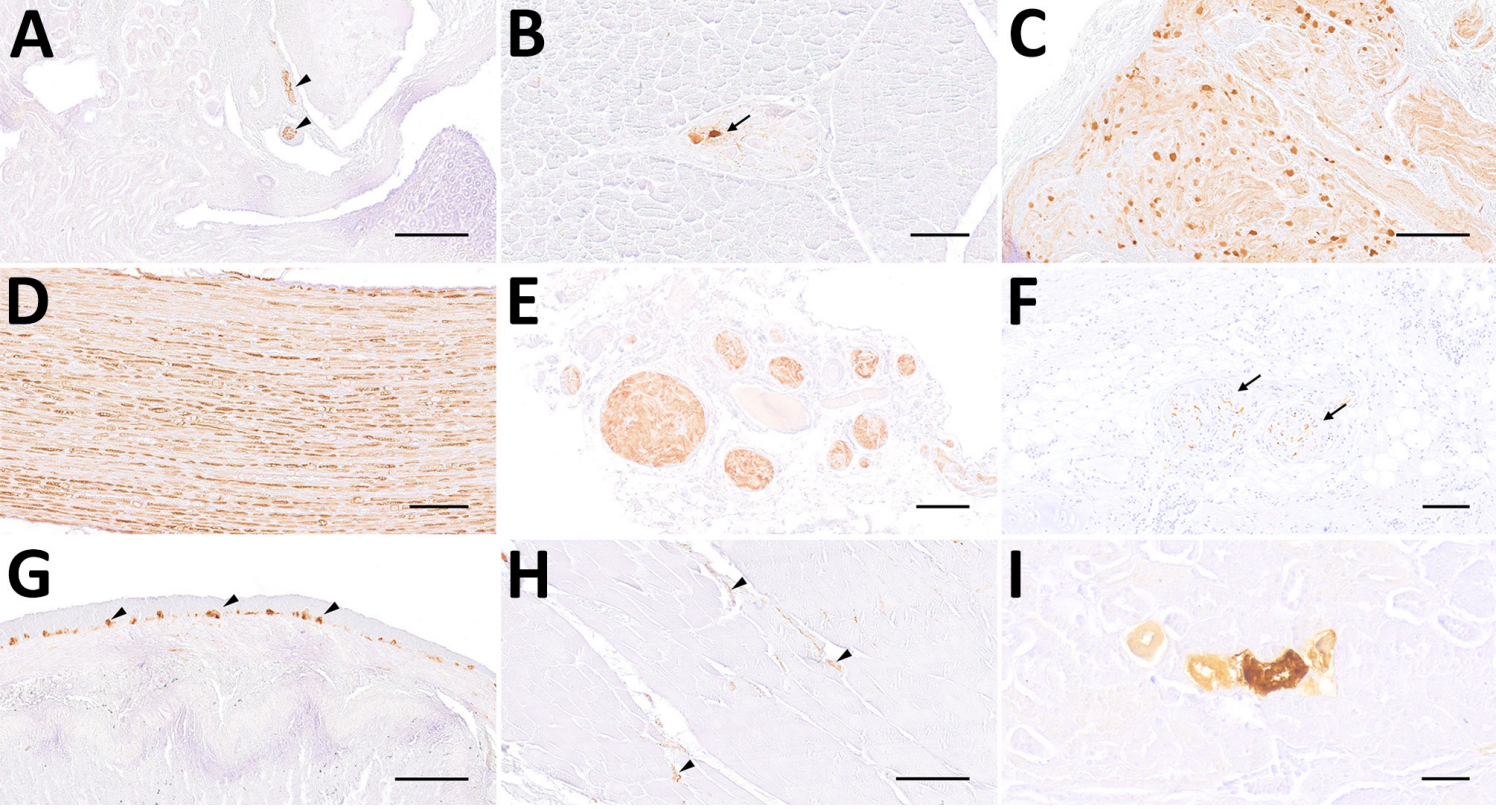
Distribution of virus antigen detected by immunohistochemistry using Bo18 antibody among peripheral organs and tissues of Borna disease virus 1–infected hedgehogs in study of the virus as cause of fatal meningoencephalomyelitis in wild hedgehogs, Germany, 2022–2025. A) Immunopositive nerve branches within the renal pelvis (arrowheads) from case 3. Scale bar represents 250 μm. B) Immunopositive ganglion neurons within salivary gland (arrow) from case 4. Scale bar represents 100 μm. C) Marked positivity within cranial mesenteric ganglion from case 3. Scale bar represents 250 μm. D) Diffusely immunopositive nerve fibers within a large fascicular nerve from case 1. Scale bar represents 100 μm. E) Large immunopositive visceral nerve branches within the mediastinum from case 2. Scale bar represents 200 μm. F) Pulmonary nerve branches with positive fibers (arrows) from case 7. Scale bar represents 100 μm. G) Widespread immunostaining of myenteric plexus (arrowheads) from case 4. Scale bar represents 250 μm. H) Immunopositive intramuscular nerve branches within the paravertebral musculature (arrowheads) from case 5. Scale bar represents 250 μm. I) A small group of immunopositive renal tubules from case 5. Scale bar represents 50 μm. All stains were 3,3′-diaminobenzidine with hematoxylin counterstain.

**Figure 7:**
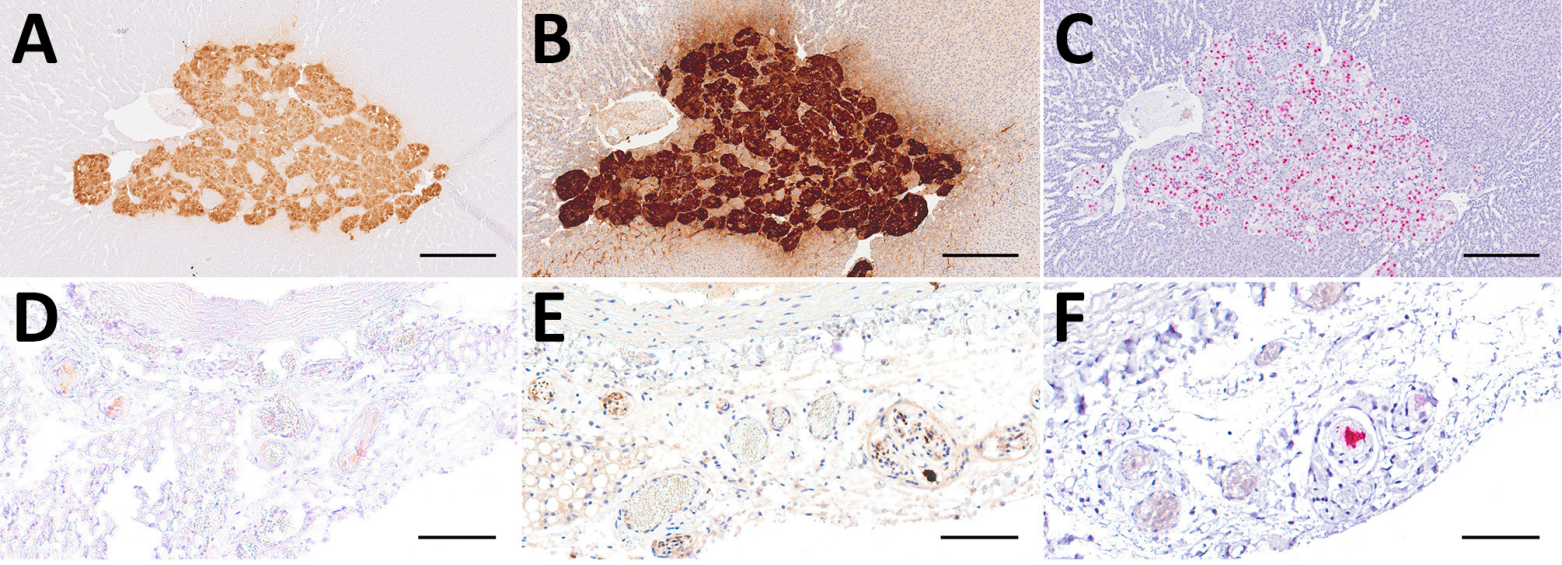
Comparison of 3 diagnostic methods, in the adrenal glands (A–C) and mediastinal nerve branches (D–F) of case 5, a hedgehog from Germany with Borna disease virus 1 (BoDV-1) infection, in study of the virus as cause of fatal meningoencephalomyelitis in wild hedgehogs, Germany, 2022–2025. Diffusely positive chromaffin cells and multifocal positive nerve branches are seen by immunohistochemistry by using BoDV-1 nucleoprotein mouse monoclonal antibody Bo18 (A, D), rabbit anti–BoDV-1 nucleoprotein polyclonal hyperimmune serum #201 (B, E) and RNAscope (Advanced Cell Diagnostics, Inc., https://acdbio.com) in situ hybridization (C, F). Scale bars: A–C, 250 μm; D–F, 100 μm. Stains: A, B, D, E, 3,3′-diaminobenzidine with hematoxylin counterstain; C, F, 2.5 HD assay–RED with hematoxylin counterstain.

We analyzed peripheral nerves and organs by IHC. Fascicular nerve roots (dorsal and ventral), dorsal root ganglia, peripheral nerves, and distal and intramural ganglia showed positive signals (Figure 4, panel D; Figure 5, panels A–H). We observed antigen-positive peripheral nerve branches and ganglia extensively across various organs (Figure 5 panels A–H; Figure 6). We detected viral antigen in ganglion and satellite cells, axons, myelin sheath, and Schwann cells of the PNS. In addition, chromaffin cells of the adrenal medulla were multifocally to diffusely strongly positive in 5 of 8 tested cases (Figure 6; Figure 7, panel A). We observed the detection of viral antigen in nonneural cells in case 5, which exhibited a strong cytoplasmic immunopositivity in a single focus of renal tubular epithelial cells (Figure 5 panel I; Figure 6).

To confirm our findings, we conducted IHC by using rabbit polyclonal hyperimmune serum #201 and RNAscope ISH for various peripheral organs from case 5. Although the staining patterns were comparable for the tested peripheral nerves and the adrenal medulla (Figure 7), we could not reproduce the IHC Bo18 signal in the tubular epithelial cells of case 5 by either of the confirmatory methods (data not shown).

### Phylogeographic Analysis of BoDV-1 Sequences from Hedgehogs

**Figure 8:**
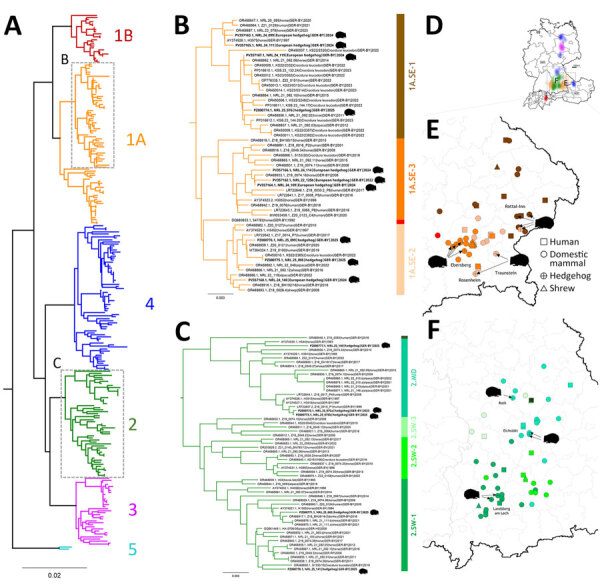
**.** Phylogeographic analysis of Borna disease virus 1 (BoDV-1) infections in hedgehogs in Bavaria in study of the virus as cause of fatal meningoencephalomyelitis in wild hedgehogs, Germany, 2022–2025. A) Phylogenetic analysis of partial genomic BoDV-1 sequences (N, X, and P genes, encoding the nucleoprotein, accessory X protein, and phosphoprotein, respectively; 1,824 nucleotides, representing genome positions 54–1,877) of all 15 BoDV-1–infected hedgehogs in combination with 258 BoDV-1 sequences from naturally infected animals and humans available from public databases (*1**,**9*). Colors of tree branches represent BoDV-1 sequence clusters 1A, 1B, and 2–5. Grey boxes mark the subtrees shown in detail in panels B and C. B, C) Detailed presentation of a subtree of cluster 1A (B) and cluster 2 (C), which contain all 15 hedgehog-derived sequences (bold). Colors of vertical bars represent subclades of cluster 1A and 2 (*1*). D) BoDV-1-endemic area in Germany, Austria, Switzerland, and Liechtenstein, adapted from Ebinger et al. (*1*). Colors represent the phylogenetic clusters shown in panel A. Licensed under a Creative Commons Attribution 4.0 International License (https://creativecommons.org/licenses/by/4.0). E, F) Detailed phylogeographic mapping of BoDV-1 cases from the phylogenetic subtree of cluster 1A (E) as shown in panel B or cluster 2 (F) as shown in panel C, to their distribution areas in Bavaria. Colors represent the different subclades. For data protection, human cases are mapped no more precisely than to the center of the administrative district of their origin.

We determined the partial genomic sequences covering the BoDV-1 N, X, and P genes (1,824 nucleotides) for all 15 BoDV-1–positive hedgehogs. Phylogenetic analysis together with 258 BoDV-1 sequences derived from public databases (*1*,*9*) revealed the hedgehog-derived sequences belonged to the BoDV-1 sequence clusters 1A or 2, which is in agreement with their origin from Bavaria (Figure 8, panels A–C). A more detailed analysis identified the hedgehog-derived sequences as belonging to subclades 1A.SE-1 (cases 1, 3, 5; Rottal-Inn, Rosenheim, Traunstein), 1A.SE-2 (case 7, 12, 13; Rottal-Inn, Rosenheim, Traunstein), 1A.SE-3 (cases 2, 4, 6, 11; Ebersberg, Germany), (Figure 8, panels B, D), 2.MID (cases 9, 10, 14; Eichstätt, Roth, Germany) and 2.SW-1 (cases 8, 15; both Landsberg am Lech) (Figure 8, panels B, C). In all cases, sequences of the same subclade derived from infected shrews, domestic mammals, or humans were found in the same or neighboring districts as the hedgehog cases (Figure 8, panels E, F).

## Discussion

Our study provides evidence of BoDV-1 infection causing meningoencephalomyelitis, radiculitis, and, in 1 case, focally extensive ganglioneuritis in wild European hedgehogs. Although BoDV-1 is known to cause fatal encephalitis in a broad range of mammalian species, well-documented cases in wild mammals are currently restricted to a single European beaver (*Castor fiber*) from 2013 (*31*). The first case of our series was detected in 2022, before 6 additional hedgehogs from the same relatively restricted area within a BoDV-1–endemic region in Bavaria, southern Germany, were submitted in 2024 within a 4-month period. In 2025, an additional 8 infected hedgehogs were submitted from a somewhat broader area in Bavaria. We cannot exclude that this temporal and regional accumulation might represent a local emergence or increase of BoDV-1 infections of hedgehogs. However, it appears possible that the initial cases raised the awareness of disease surveillance centers, leading to more frequent diagnosis of a previously underreported entity.

The relatively frequent detection of BoDV-1 in hedgehogs suggests hedgehogs might be particularly susceptible to BoDV-1 infection, possibly because of their biological relationship to shrews, the reservoir hosts of BoDV-1 that the virus is adapted to (*20*). This possibility raised the question whether infected hedgehogs might serve only as spillover dead-end hosts that develop disease without viral shedding or whether they also show broad viral tissue distribution, viral shedding, or even an asymptomatic infection, similar to BoDV-1 reservoir hosts (*11*). A similar intermediate role has been described for psittacines affected by parrot bornaviruses 1−8 (species *Orthobornavirus alphapsittaciforme* and *O*. *betapsittaciforme*). Affected birds suffer from neurologic disease and can transmit the virus to a broad range of other psittaciformes originating from different continents and are therefore unlikely to all represent original reservoir hosts of those viruses (*32*).

To date, neurological signs induced by a lymphoplasmohistiocytic meningoencephalitis developed in all BoDV-1–positive hedgehogs, similar to the case for spillover hosts such as horses, alpacas, sheep, and humans (*28*–*30*). Because this finding might be biased by our initial case selection focusing on animals with neurologic abnormalities, we extended the study to nonencephalitic hedgehogs from endemic areas. We could not detect BoDV-1 in brains of 33 nonencephalitic hedgehogs. However, a larger survey of neurologically inconspicuous hedgehogs in BoDV-1–endemic areas is required to rule out the possibility of mild or asymptomatic BoDV-1 infections.

Of note, the BoDV-1–infected hedgehogs in our study exhibit differences from other affected species in terms of lesion and virus distribution spatial characteristics within the CNS. In horses, the most prominently affected CNS region is the hippocampal formation, followed by limbic system, basal ganglia, and brainstem (*29*,*30*). Humans seem to consistently show virus infestation hotspots in the brainstem and telencephalon or diencephalon (*28*). We did not observe the same distribution characteristics in the hedgehogs. Instead, we observed a uniform distribution of inflammatory infiltrates, virus antigen, and RNA throughout the entire CNS. Beyond neuronal infection, both IHC and ISH revealed a prominent infection of glial cells and Schwann cells, whereas viral RNA was also detected in ependymal cells of 12 animals (Figure 1). A similar cell tropism was previously described in the CNS of humans (*28*) and experimentally infected rats (*33*,*34*). In the studied hedgehogs, inflammation was mainly restricted to the CNS and spinal nerve roots, whereas only 1 animal showed ganglioneuritis of the cranial mesenteric ganglion. To our knowledge, distal ganglioneuritis is not a typical feature in naturally BoDV-1–infected horses or alpacas (*8*,*29*), but it is a hallmark of parrot bornavirus infection in parrots (*32*). Inflammation in peripheral nerves has been described for a few BoDV-1–infected human patients, whose illness manifested with Guillain-Barré–like neuropathy at early stages of infection (*5*,*28*,*35*).

Detection of BoDV-1 antigen or RNA in peripheral nerves and ganglia has been sporadically described also for alpacas and human patients as well as a BoDV-1–infected beaver (*28*,*29*,*31*). That finding has been discussed mainly as representing centrifugal virus dissemination from the brain, as experimentally shown for mice and rats (*36*,*37*). Compared with those species, BoDV-1–positive cells in the PNS were surprisingly common in the analyzed hedgehogs; we identified positive nerve fibers and ganglion cells in several organs for each of them.

In 5 animals, viral antigen was seen in chromaffin cells of the adrenal medulla, whereas in 1 animal we observed focal cytoplasmic immunopositivity in the focus of renal tubular epithelial cells. Although the cytoplasmic immunopositivity could not be confirmed by IHC by using another BoDV-1 N antibody or by RNAscope ISH, our concern is that individual BoDV-1–infected hedgehogs might shed the virus via mucosal surfaces. Unfortunately, urine, fecal, and mucosal swab samples were not available from this animal. However, we emphasize that the extent of viral presence on the epithelial surfaces of this single animal is much lower compared with infected bicolored white-toothed shrews, in which viral antigen is usually found widespread on various epithelial surfaces (*11*). We detected BoDV-1 RNA in oral swab and urine samples of 2 hedgehogs at levels barely above the assay detection limit. We do not know if those low amounts of viral RNA represent shedding of infectious virus.

Most BoDV-1–infected hedgehogs displayed neurologic manifestations before or shortly after capture. Only case 12 had clinical signs >6 months after admission, indicating potential infection in the rescue center, given the assumed incubation period of several weeks to few months (*1*). However, it remains unknown if the hibernation-related metabolic suppression could delay the onset of this immune-mediated disease. The BoDV-1 sequences found in the 15 cases belonged to 5 different phylogenetic subclades, all reflecting the dominant virus types in the region in which each animal was found (*1*). That result argues for individual spillover events from the local shrew reservoir, rather than for a hedgehog-adapted BoDV-1 variant circulating in their populations.

In summary, we present a series of BoDV-1 cases in European hedgehogs. BoDV-1 infections might be greatly underreported in this species and wild mammals in general. It is therefore essential to consider BoDV-1 infection as a possible differential diagnosis in hedgehogs with CNS signs and encephalitic lesions in endemic regions, even if Joest-Degen inclusion bodies are not present. Despite being distant relatives of bicolored white-toothed shrews, the identified BoDV-1–infected hedgehogs showed the signature of typical spillover dead-end hosts, with fatal lymphoplasmohistiocytic encephalitis and an almost exclusively neurotropic infection. However, the broader viral presence across the PNS of hedgehogs and occasional detection of viral antigen in nonneural cells, possibly including renal epithelial cells in 1 animal, raise concerns if singular infected hedgehogs might shed virus. In such cases, the amount of excreted virus would likely be considerably lower than for regular reservoir hosts. Moreover, BoDV-1 spillover transmission to humans appears to be generally inefficient; only a few cases occur per year even in areas where the virus is endemic in the local shrew population (*1*,*9*). However, given the potentially close contact of humans and hedgehogs and the high case-fatality rate of zoonotic BoDV-1 infections, our results not only call for further investigations into the epidemiology of BoDV-1 infections in hedgehogs but also emphasize that standard hygiene measures should be implemented whenever handling hedgehogs, particularly for those with neurologic disorders.

Appendix Additional information about Borna disease virus 1 cause of fatal meningoencephalomyelitis in wild hedgehogs, Germany, 2022–2025.

## References

[1] Ebinger A, Santos PD, Pfaff F, Dürrwald R, Kolodziejek J, Schlottau K, et al. Lethal Borna disease virus 1 infections of humans and animals—in-depth molecular epidemiology and phylogeography. Nat Commun. 2024;15:7908. 10.1038/s41467-024-52192-x 39256401 PMC11387626

[2] Malbon AJ, Dürrwald R, Kolodziejek J, Nowotny N, Kobera R, Pöhle D, et al. New World camelids are sentinels for the presence of Borna disease virus. Transbound Emerg Dis. 2022;69:451–64. 10.1111/tbed.14003 33501762

[3] Schulze V, Große R, Fürstenau J, Forth LF, Ebinger A, Richter MT, et al. Borna disease outbreak with high mortality in an alpaca herd in a previously unreported endemic area in Germany. Transbound Emerg Dis. 2020;67:2093–107. 10.1111/tbed.13556 32223069

[4] Vahlenkamp TW, Konrath A, Weber M, Müller H. Persistence of Borna disease virus in naturally infected sheep. J Virol. 2002;76:9735–43. 10.1128/JVI.76.19.9735-9743.2002 12208952 PMC136490

[5] Schlottau K, Forth L, Angstwurm K, Höper D, Zecher D, Liesche F, et al. Fatal encephalitic Borna disease virus 1 in solid-organ transplant recipients. N Engl J Med. 2018;379:1377–9. 10.1056/NEJMc1803115 30281984

[6] Korn K, Coras R, Bobinger T, Herzog SM, Lücking H, Stöhr R, et al. Fatal encephalitis associated with Borna disease virus 1. N Engl J Med. 2018;379:1375–7. 10.1056/NEJMc1800724 30281979

[7] Niller HH, Angstwurm K, Rubbenstroth D, Schlottau K, Ebinger A, Giese S, et al. Zoonotic spillover infections with Borna disease virus 1 leading to fatal human encephalitis, 1999–2019: an epidemiological investigation. Lancet Infect Dis. 2020;20:467–77. 10.1016/S1473-3099(19)30546-8 31924550

[8] Weissenböck H, Bagó Z, Kolodziejek J, Hager B, Palmetzhofer G, Dürrwald R, et al. Infections of horses and shrews with Bornaviruses in upper Austria: a novel endemic area of Borna disease. Emerg Microbes Infect. 2017;6:e52. 10.1038/emi.2017.36 28634359 PMC5520313

[9] Böhmer MM, Haring VC, Schmidt B, Saller FS, Coyer L, Chitimia-Dobler L, et al. One Health in action: investigation of the first detected local cluster of fatal borna disease virus 1 (BoDV-1) encephalitis, Germany 2022. J Clin Virol. 2024;171:105658. 10.1016/j.jcv.2024.105658 38447459

[10] Rubbenstroth D, Schlottau K, Schwemmle M, Rissland J, Beer M. Human bornavirus research: back on track! PLoS Pathog. 2019;15:e1007873. 10.1371/journal.ppat.1007873 31369648 PMC6675037

[11] Nobach D, Bourg M, Herzog S, Lange-Herbst H, Encarnação JA, Eickmann M, et al. Shedding of infectious Borna disease virus-1 in living bicolored white-toothed shrews. PLoS One. 2015;10:e0137018. 10.1371/journal.pone.0137018 26313904 PMC4552160

[12] Hilbe M, Herrsche R, Kolodziejek J, Nowotny N, Zlinszky K, Ehrensperger F. Shrews as reservoir hosts of Borna disease virus. Emerg Infect Dis. 2006;12:675–7. 10.3201/eid1204.051418 16704819 PMC3294707

[13] Dürrwald R, Kolodziejek J, Weissenböck H, Nowotny N. The bicolored white-toothed shrew Crocidura leucodon (HERMANN 1780) is an indigenous host of mammalian Borna disease virus. PLoS One. 2014;9:e93659. 10.1371/journal.pone.0093659 24699636 PMC3974811

[14] Riley PY, Chomel BB. Hedgehog zoonoses. Emerg Infect Dis. 2005;11:1–5. 10.3201/eid1101.040752 15705314 PMC3294334

[15] Ruszkowski JJ, Hetman M, Turlewicz-Podbielska H, Pomorska-Mól M. Hedgehogs as a potential source of zoonotic pathogens—a review and an update of knowledge. Animals (Basel). 2021;11:1754. 10.3390/ani11061754 34208276 PMC8230866

[16] Schönbächler K, Hatt J, Silaghi C, Merz N, Fraefel C, Bachofen C. Confirmation of tick-borne encephalitis virus in a European hedgehog (*Erinaceus europaeus*) [in German]. Schweiz Arch Tierheilkd. 2019;161:23–31. 10.17236/sat00191 30602429

[17] Faragó Z. Rabid hedgehog in inner-city area of Budapest [in Hungarian]. Orv Hetil. 1997;138:2231–2. 9333732

[18] Duque-Valencia J, Sarute N, Olarte-Castillo XA, Ruíz-Sáenz J. Evolution and interspecies transmission of canine distemper virus—an outlook of the diverse evolutionary landscapes of a multi-host virus. Viruses. 2019;11:582. 10.3390/v11070582 31247987 PMC6669529

[19] Matiasek K, Pfaff F, Weissenböck H, Wylezich C, Kolodziejek J, Tengstrand S, et al. Mystery of fatal ‘staggering disease’ unravelled: novel rustrela virus causes severe meningoencephalomyelitis in domestic cats. Nat Commun. 2023;14:624. 10.1038/s41467-023-36204-w 36739288 PMC9899117

[20] Douady CJ, Chatelier PI, Madsen O, de Jong WW, Catzeflis F, Springer MS, et al. Molecular phylogenetic evidence confirming the Eulipotyphla concept and in support of hedgehogs as the sister group to shrews. Mol Phylogenet Evol. 2002;25:200–9. 10.1016/S1055-7903(02)00232-4 12383761

[21] Steininger P, Ensser A, Knöll A, Korn K. Results of tick-borne encephalitis virus (TBEV) diagnostics in an endemic area in southern Germany, 2007 to 2022. Viruses. 2023;15:2357. 10.3390/v15122357 38140598 PMC10748111

[22] Thilén E, Rubbenstroth D, Tengstrand S, Pfaff F, Wensman JJ, Ley C. Evidence of rustrela virus-associated feline staggering disease in Sweden since the 1970s. Acta Vet Scand. 2024;66:59. 10.1186/s13028-024-00783-5 39580473 PMC11585236

[23] Li Z, Feng Z, Ye H. Rabies viral antigen in human tongues and salivary glands. J Trop Med Hyg. 1995;98:330–2. 7563261

[24] Haines DM, Martin KM, Chelack BJ, Sargent RA, Outerbridge CA, Clark EG. Immunohistochemical detection of canine distemper virus in haired skin, nasal mucosa, and footpad epithelium: a method for antemortem diagnosis of infection. J Vet Diagn Invest. 1999;11:396–9. 10.1177/104063879901100502 12968751

[25] Haas B, Becht H, Rott R. Purification and properties of an intranuclear virus-specific antigen from tissue infected with Borna disease virus. J Gen Virol. 1986;67:235–41. 10.1099/0022-1317-67-2-235 3080548

[26] Wang F, Flanagan J, Su N, Wang L-C, Bui S, Nielson A, et al. RNAscope: a novel in situ RNA analysis platform for formalin-fixed, paraffin-embedded tissues. J Mol Diagn. 2012;14:22–9. 10.1016/j.jmoldx.2011.08.002 22166544 PMC3338343

[27] Garman RH. Histology of the central nervous system. Toxicol Pathol. 2011;39:22–35. 10.1177/0192623310389621 21119051

[28] Liesche F, Ruf V, Zoubaa S, Kaletka G, Rosati M, Rubbenstroth D, et al. The neuropathology of fatal encephalomyelitis in human Borna virus infection. Acta Neuropathol. 2019;138:653–65. 10.1007/s00401-019-02047-3 31346692 PMC6778062

[29] Fürstenau J, Richter MT, Erickson NA, Große R, Müller KE, Nobach D, et al. Borna disease virus 1 infection in alpacas: comparison of pathological lesions and viral distribution to other dead-end hosts. Vet Pathol. 2024;61:62–73. 10.1177/03009858231185107 37431864

[30] Bilzer T, Planz O, Lipkin WI, Stitz L. Presence of CD4+ and CD8+ T cells and expression of MHC class I and MHC class II antigen in horses with Borna disease virus-induced encephalitis. Brain Pathol. 1995;5:223–30. 10.1111/j.1750-3639.1995.tb00598.x 8520721

[31] Ellenberger C, Heenemann K, Vahlenkamp TW, Grothmann P, Herden C, Heinrich A. Borna disease in an adult free-ranging Eurasian beaver (Castor fiber albicus). J Comp Pathol. 2024;209:31–5. 10.1016/j.jcpa.2024.01.003 38350270

[32] Rubbenstroth D. Avian Bornavirus research—a comprehensive review. Viruses. 2022;14:1513. 10.3390/v14071513 35891493 PMC9321243

[33] Carbone KM, Moench TR, Lipkin WI. Borna disease virus replicates in astrocytes, Schwann cells and ependymal cells in persistently infected rats: location of viral genomic and messenger RNAs by in situ hybridization. J Neuropathol Exp Neurol. 1991;50:205–14. 10.1097/00005072-199105000-00003 2022964

[34] Werner-Keišs N, Garten W, Richt JA, Porombka D, Algermissen D, Herzog S, et al. Restricted expression of Borna disease virus glycoprotein in brains of experimentally infected Lewis rats. Neuropathol Appl Neurobiol. 2008;34:590–602. 10.1111/j.1365-2990.2008.00940.x 18282160

[35] Coras R, Korn K, Kuerten S, Huttner HB, Ensser A. Severe bornavirus-encephalitis presenting as Guillain-Barré-syndrome. Acta Neuropathol. 2019;137:1017–9. 10.1007/s00401-019-02005-z 30953131

[36] Shankar V, Kao M, Hamir AN, Sheng H, Koprowski H, Dietzschold B. Kinetics of virus spread and changes in levels of several cytokine mRNAs in the brain after intranasal infection of rats with Borna disease virus. J Virol. 1992;66:992–8. 10.1128/jvi.66.2.992-998.1992 1731117 PMC240801

[37] Enbergs HK, Vahlenkamp TW, Kipar A, Müller H, Haimo K. Enbergs, Thomas W Vahlenkam. Experimental infection of mice with Borna disease virus (BDV): replication and distribution of the virus after intracerebral infection. J Neurovirol. 2001;7:272–7. 10.1080/13550280152403317 11517401

