# Supplementary material for: Borna Disease Virus 1 as Cause of Fatal Meningoencephalomyelitis in Wild Hedgehogs, Germany, 2022–2025
Source: Emerg Infect Dis. 2026 May;32(5):675–86. doi: 10.3201/eid3205.250952 (PMC13174970; doi:10.3201/eid3205.250952)
Supplement: Appendix — Additional information about Borna disease virus 1 cause of fatal meningoencephalomyelitis in wild hedgehogs, Germany, 2022–2025. [file 25-0952-Techapp-s1.pdf]

Article DOI: https://doi.org/10.3201/eid3205.250952

EID cannot ensure accessibility for supplementary materials supplied by authors. Readers who have difficulty accessing supplementary content should contact the authors for assistance.

Borna Disease Virus 1 as Cause of Fatal Meningoencephalomyelitis in Wild Hedgehogs, Germany, 2022–2025

Appendix

A. Appendix tables

Appendix Table 1. European hedgehogs tested for BoDV-1 in this study

Lymphohistiocytic encephalitis	Origin from BoDV-1-endemic area	Years	Number of tested animals	Number of BoDV-1 RT-qPCR-positive animals (%)
Yes	Yes	2022–2025	16	15 (94%)
No	Yes	2024–2025	33	0

Appendix Table 2. Primers and probes used for RT-qPCR testing and sequencing of partial BoDV-1 genomes

Assay	Primer/Probe name	Sequence (5′ to 3′)	Reference
BoDV-1 Mix-1	BoDV-1_1258+	TAGTYAGGAGGCTCAATGGCA	(*1*)
	BoDV-1_1316_FAM	FAM-AAGAAGATCCCCAGACACTACGACG-BHQ1	(*1*)
	BoDV-1_1419-	GTCCYTCAGGAGCTGGTC	(*1*)
BoDV-1 Mix-6	BoDV-1_2231+	CAATYAATGCAGCYTTCAATGTCTT	(*1*)
	BoDV-1_2285as_FAM	FAM-CCARCACCAATGTTCCGAAGCCG-BHQ1	(*1*)
	BoDV-1_2305-	GAATGTCYGGGCCGAGAG	(*1*)
panRusV-2a	RusV_234+	CCCCGTGTTCCTAGGCAC	(*2*)
	RusV_256_P	FAM-GTGAGCGACCACCCAGCACTCCA-BHQ1	(*2*)
	RusV_323-	TCGCCCCATTCWACCCAATT	(*2*)
TBE1-Mix	TBEV_11054+	GGGCGGTTCTTGTTCTCC	(*3*)
	TBEV_11073_P	FAM-TGAGCCACCATCACCCAGACACA-BHQ1	(*3*)
	TBEV_11121-	ACACATCACCTCCTTGTCAGACT	(*3*)
β actin mix 2*	ACT_F_1005–1029	CAGCACAATGAAGATCAAGATCATC	(*4*)
	ACT_P_1081–1105_HEX	HEX-TCGCTGTCCACCTTCCAGCAGATGT-BHQ1	(*4*)
	ACT_R_1135–1114	CGGACTCATCGTACTCCTGCTT	(*4*)
eGFP mix 1*	EGFP-1-F	GACCACTACCAGCAGAACAC	(*5*)
	EGFP-Probe1_HEX	HEX-AGCACCCAGTCCGCCCTGAGCA-BHQ1	(*5*)
	EGFP-2-R	GAACTCCAGCAGGACCATG	(*5*)
BoDV-1 amplification for sequencing	PaBV-2_1+	TGTTGCGGTAACAACCAAC	(*6*)
	BoDV-1_1161-	TTAGACCAGTCACACCTATC	(*7*)
	BoDV-1_1068+	GTATAGGCGCCGCGAGATAT	(*7*)
	BoDV-1_2311-	AAGATCGAATGTCTGGGCCG	(*7*)
sequencing primers	BoDV-1_523+	GCAGGAGCCGARCAGATCAAG	(*7*)
	BoDV-1_656-	GGTTGGCCGTTAATCCAATC	(*7*)
	BoDV-1_1621+	GAAACCATCCAGACAGCTCAG	(*7*)
	BoDV-1_1816-	GAGGTGCAGGATGGGAGGG	(*7*)

*Control reactions for the detection of host RNA (β actin) or an external RNA spiked into the sample during RNA extraction (eGFP).

Appendix Table 3. Antibodies used for immunohistochemistry (IHC)

Name	Host, type	Target*	Source	Dilution factor	Reference†
Bo18	mouse, monoclonal	BoDV-1 N	antibody collection of the FLI (established by Lothar Stitz)	1:200	(*8*)
#201	rabbit, polyclonal	BoDV-1 N	Institute for Virology, University Medical Center Freiburg, Germany	1:5000	(*9*)
CDV	mouse, monoclonal	CDV	VMRD, Pullman, WA, USA	1:200	
CD3	rabbit, polyclonal	CD3	Dako Denmark A/S, Denmark	1:200	
GFAP	rabbit, polyclonal	GFAP	Dako Denmark A/S, Denmark	1:100	
Iba1	rabbit, polyclonal	Iba1	Abcam, USA	1:200	
Pax5	mouse, monoclonal	Pax5	Biozol, Germany	1:150	
RV	mouse, monoclonal	RV N	antibody collection of the institute of veterinary pathology of LMU	1:200	(*10*)

*BoDV-1 N: Borna disease virus 1 nucleoprotein; CDV: canine distemper virus; CD3: cluster of differentiation 3; GFAP: glial fibrillary acidic protein; Iba1: Ionized calcium binding adaptor molecule 1; Pax5: Paired box protein 5; RVN: Rabies virus nucleocapsin (N)

†References are provided for non-commercially available antibodies. Commercial antibodies were used according to the manufacturers' specifications.

Appendix Table 4. Additional information on BoDV-1-infected European hedgehogs included in this study

Characteristics	Case 1	Case 2	Case 3	Case 4	Case 5	Case 6	Case 7	Case 8	Case 9	Case 10	Case 11	Case 12	Case 13	Case 14	Case 15
Weight (g)	960	800	830	640	480	542	784	860	402	472	784	700	400	860	659
Sex	M	M	M	M	F	F	M	F	M	F	M	F	F	F	F
Mode of death*	E	D	E	E	E	E	E	D	E	E	E	E	E	E	E
Neurologic signs															
Incoordination	x		x	x	x	x	x		x	x	x				x
Gait abnormalities	x	x		x		x	x	x	x	x	x				x
Seizures		x						x				x	x	x	
Head tilt				x											
Twitching		x			x										
Apathy		x		x		x									
Impaired thermo-regulation				x		x									
Therapy															
Treatment attempts†	Yes	No	NS	Yes	NS	Yes	NS	Yes	NS	NS	Yes	NS	Yes	Yes	Yes

*Mode of death: E = euthanized; D = spontaneous death.
†Treatment refers to supportive or empirical therapy (e.g., antibiotics, corticosteroids, insecticides, and/or anthelmintics), NS, not specified.

B. Detailed description of immunohistochemical procedures

The detailed immunohistochemical procedures for all used antibodies are described below. All antibodies had been tested and optimized for the use in this species in a pilot trial (data not shown). Immunohistochemistry for GFAP, CD3, Iba1, BoDV-1 (monoclonal antibody Bo18), rabies virus, canine distemper virus (CDV) and Pax5 was performed manually, while for BoDV-1 (rabbit polyclonal hyperimmune serum #201) an automated immunostainer was employed. For details on the antibodies see Appendix table S3.

B.1. Manual immunohistochemistry:

I. Deparaffinization and rehydration

Sections were deparaffinized in xylene and rehydrated in descending ethanol series followed by rinsing with distilled water or Tris-buffered saline (TBS).

II. Antigen retrieval

Antigen retrieval was performed through heat-induced epitope retrieval (HIER) using a microwave, with an addition of Tris-EDTA buffer (pH 9.0) for CD3, an addition of citrate buffer (pH 6.0) for GFAP, Iba1, CDV and Pax5, and without pre-treatment for Bo18, while no antigen retrieval was performed for rabies virus. After the microwave, the sections were let to cool and then rinsed with distilled water or TBS.

III. Blocking Endogenous Peroxidase Activity

Endogenous peroxidase activity was quenched by incubating sections in 3% hydrogen peroxide (H_2O_2), followed by rinsing with TBS.

IV. Blocking Non-Specific Binding

Non-specific binding was blocked using ImPRESS Normal Horse Serum for GFAP, a stock solution comprising blocking buffer with goat serum (1:40 dilution) supplemented with Avidin for CD3 and CDV, a stock solution comprising blocking buffer with rabbit serum (1:40 dilution) supplemented with Avidin for Iba1, normal goat serum (1:20 dilution) for Bo18 and rabies virus.

Blocking buffer refers to a solution of TBS prepared by diluting a 10X TBS stock 1:10 with distilled water to obtain a 1X TBS working solution, with addition of Bovine Serum Albumin (BSA) (1%), Triton X-100 (0.1%), of Goldfish Gelatin (0.2%) and of a 10% solution of Sodium Azide (0.02%).

V. Primary Antibody Incubation

Sections were incubated with the primary antibodies as follows:

1. Rabbit polyclonal anti-GFAP antibody (Dako Denmark A/S, Denmark) in 1:800 dilution overnight at 4°C, diluted in blocking buffer.

2. Rabbit polyclonal anti-CD3 antibody (Dako Denmark A/S, Denmark) in 1:100 dilution for 1 hour at room temperature, diluted in stock solution (blocking buffer and goat serum in 1:40 dilution) supplemented with Biotin.

3. Mouse monoclonal Bo18 antibody (1:1,000 dilution) for 1 hour at room temperature, diluted in TBS.

4. Rabbit polyclonal anti-Iba1 antibody (Abcam, USA) in 1:500 dilution overnight at 4°C, diluted in blocking buffer supplemented with Biotin.

5. Mouse monoclonal anti-CDV antibody (VMRD, Pullman, WA, USA) in 1:1,000 dilution overnight at 4°C, diluted in stock solution (blocking buffer and goat serum in 1:40 dilution) supplemented with Biotin.

6. Mouse monoclonal anti-rabies virus antibody at a 1:200 dilution overnight at 4°C, diluted in TBS.

7. Mouse monoclonal anti-PAX5 antibody (Biozol, Germany) in 1:150 dilution overnight at 4°C, diluted in TBS.

All sections were subsequently rinsed with TBS.

VI. Secondary Antibody Incubation

Sections were incubated with the secondary antibodies as follows:

1. GFAP: Sections were incubated with ImPRESS HRP anti-rabbit Polymer Kit for 30 minutes.

2. CD3: Sections were incubated with biotinylated goat anti-rabbit secondary antibody (1:200 dilution) for 50 minutes.

3. Bo18: Sections were incubated with biotinylated goat anti-mouse secondary antibody (1:200 dilution) for 50 minutes.

4. Iba1: Sections were incubated with biotinylated goat anti-rabbit secondary antibody (1:200 dilution) for 1 hour.

5. CDV: Sections were incubated with biotinylated goat anti-mouse secondary antibody (1:200 dilution) for 50 minutes.

6. Rabies: Sections were incubated with biotinylated goat anti-mouse secondary antibody (1:200 dilution) for 1 hour.

7. Pax5: Sections were incubated with ImPRESS HRP anti-mouse Polymer Kit for 30 minutes.

All sections were subsequently rinsed in TBS.

VII. ABC Complex

All sections except for GFAP and Pax5 were incubated with ABC reagent (Avidin-Biotin Complex), in a 1:100 dilution for 30 minutes, and subsequently rinsed in TBS.

VIII. Detection, Counterstaining, Dehydration and Mounting

Chromogenic detection was performed using 3,3′-diaminobenzidine (DAB) substrate. Counterstaining was performed using hematoxylin and sections were then rinsed in tap water. Afterwards dehydration was performed with an ascending ethanol series, sections were then cleared in xylene, and coverslipped using a permanent mounting medium.

IX. Positive controls

Sections of hedgehog brain (GFAP), hedgehog lymph node (CD3, Iba1 and Pax5), BoDV-1-infected horse brain (Bo18), rabies virus-infected dog brain (rabies Virus) and CDV-infected dog brain (CDV) were used as positive controls for the staining.

X. Negative controls

Negative controls were performed using species-matched irrelevant antibodies. A rabbit anti-calcitonin antibody was used as a negative control for immunohistochemical markers employing rabbit primary antibodies, and a mouse anti–feline coronavirus antibody was used for markers employing mouse primary antibodies.

XI. Evaluation and interpretation

Distinctive multiple and coalescing intracellular DAB-brown intracellular dots, as observed in the positive controls, were interpreted as positive staining.

B.2. Automated immunohistochemistry:

Immunohistochemistry for BoDV-1 rabbit polyclonal hyperimmune serum #201 was performed using a fully automated staining system (BOND-III; Leica Biosystems, Wetzlar, Germany). Antigen retrieval was performed through heat-induced epitope retrieval (HIER), in

EDTA buffer at 100°C for 100 minutes. The slides were incubated with the primary antibody (polyclonal, rabbit, dilution 1:5000) for 32 minutes. Antibody detection and counterstaining was performed through the BOND Polymer Refine Detection Kit (Leica Biosystems, Germany). A positive controls of human BoDV-1 infected brain, was used to confirm antibody specificity.

C. Concurrent pathologies

Eleven of the 15 BoDV-1-positive hedgehogs histologically showed concurrent inflammatory changes of the lungs, ranging from mild lymphoplasmacytic interstitial pneumonia to lymphoplasmacytic and histiocytic peribronchitis, bronchopneumonia and pleuritis, sometimes associated with intralesional nematodes, morphologically most consistent with *Eucoleus aerophilus* (syn. *Capillaria aerophila*) (*11*). Moreover, 9 animals showed a mild to moderate, multifocal, lymphoplasmacytic interstitial nephritis and one animal showed minimal lymphoplasmacytic pyelitis of unclear origin. Two animals showed mild to moderate lymphoplasmacytic enteritis or gastroenteritis. Lesions seen in single animals include mild periportal lymphoplasmacytic hepatitis, mild lymphocytic pancreatitis and adrenocortical necrosis. In all cases, in which spleen was sampled, there was prominent extramedullary hematopoiesis, a finding common to this species (*12*).

References

1. Schlottau K, Forth L, Angstwurm K, Höper D, Zecher D, Liesche F, et al. Fatal encephalitic Borna disease virus 1 in solid-organ transplant recipients. N Engl J Med. 2018;379:1377–9. PubMed https://doi.org/10.1056/NEJMc1803115

2. Thilén E, Rubbenstroth D, Tengstrand S, Pfaff F, Wensman JJ, Ley C. Evidence of rustrela virus-associated feline staggering disease in Sweden since the 1970s. Acta Vet Scand. 2024;66:59. PubMed https://doi.org/10.1186/s13028-024-00783-5

3. Schwaiger M, Cassinotti P. Development of a quantitative real-time RT-PCR assay with internal control for the laboratory detection of tick borne encephalitis virus (TBEV) RNA. J Clin Virol. 2003;27:136–45. PubMed https://doi.org/10.1016/S1386-6532(02)00168-3

4. Toussaint JF, Sailleau C, Breard E, Zientara S, De Clercq K. Bluetongue virus detection by two real-time RT-qPCRs targeting two different genomic segments. J Virol Methods. 2007;140:115–23. PubMed https://doi.org/10.1016/j.jviromet.2006.11.007

5. Hoffmann B, Depner K, Schirrmeier H, Beer M. A universal heterologous internal control system for duplex real-time RT-PCR assays used in a detection system for pestiviruses. J Virol Methods. 2006;136:200–9. PubMed https://doi.org/10.1016/j.jviromet.2006.05.020

6. Rubbenstroth D, Schmidt V, Rinder M, Legler M, Twietmeyer S, Schwemmer P, et al. Phylogenetic analysis supports horizontal transmission as a driving force of the spread of avian bornaviruses. PLoS One. 2016;11:e0160936. PubMed https://doi.org/10.1371/journal.pone.0160936

7. Schulze V, Große R, Fürstenau J, Forth LF, Ebinger A, Richter MT, et al. Borna disease outbreak with high mortality in an alpaca herd in a previously unreported endemic area in Germany. Transbound Emerg Dis. 2020;67:2093–107. PubMed https://doi.org/10.1111/tbed.13556

8. Haas B, Becht H, Rott R. Purification and properties of an intranuclear virus-specific antigen from tissue infected with Borna disease virus. J Gen Virol. 1986;67:235–41. PubMed https://doi.org/10.1099/0022-1317-67-2-235

9. Zimmermann V, Rinder M, Kaspers B, Staeheli P, Rubbenstroth D. Impact of antigenic diversity on laboratory diagnosis of avian bornavirus infections in birds. J Vet Diagn Invest. 2014;26:769–77. PubMed https://doi.org/10.1177/1040638714547258

10. Li Z, Feng Z, Ye H. Rabies viral antigen in human tongues and salivary glands. J Trop Med Hyg. 1995;98:330–2. PubMed

11. Lehmann S, Dervas E, Ruiz Subira A, Eulenberger U, Gimmel A, Grimm F, et al. Verminous pneumonia in European hedgehogs (*Erinaceus europaeus*). Vet Pathol. 2024;61:256–68. PubMed https://doi.org/10.1177/03009858231193103

12. Zacharopoulou M, Guillaume E, Coupez G, Bleuart C, Le Loc'h G, Gaide N. Causes of mortality and pathological findings in European hedgehogs (*Erinaceus europaeus*) admitted to a wildlife care centre in southwestern France from 2019 to 2020. J Comp Pathol. 2022;190:19–29. PubMed https://doi.org/10.1016/j.jcpa.2021.11.001
